# Effects of mindfulness-based cognitive therapy for Chinese adults with PTSD symptoms: protocol for a randomised controlled trial

**DOI:** 10.1186/s12888-024-05840-x

**Published:** 2024-05-29

**Authors:** Bertha Sze Wing Mak, Dexing Zhang, Candice Ling Yuet Man Powell, Maria Kwan Wa Leung, Herman Hay Ming Lo, Xue Yang, Benjamin Hon Kei Yip, Eric Kam Pui Lee, Zijun Xu, Samuel Yeung Shan Wong

**Affiliations:** 1 Faculty of Education, The Chinese University of Hong Kong, Hong Kong, China; 2 JC School of Public Health and Primary Care, The Chinese University of Hong Kong, Hong Kong, China; 3 Mind HK, Hong Kong, China; 4 Department of Family Medicine & Primary Health Care, New Territories East Cluster, Hospital Authority, Hong Kong, China; 5 Department of Applied Social Sciences, The Hong Kong Polytechnic University, Hong Kong, China

**Keywords:** PTSD, PTSD symptoms, Mindfulness, Mindfulness-based cognitive therapy, Seeking safety, Randomized controlled trial

## Abstract

**Introduction:**

Emerging evidence supports mindfulness as a potential psychotherapy for post-traumatic stress disorder (PTSD). Individuals with subthreshold PTSD experience significant impairment in their daily life and functioning due to PTSD symptoms, despite not meeting the full diagnostic criteria for PTSD in DSM-5. Mindfulness skills, including non-judgmental acceptance, attentional control and openness to experiences may help alleviate PTSD symptoms by targeting characteristics such as intensified memory processing, dysregulated hyperarousal, avoidance, and thought suppression. This trial aims to test the effects of mindfulness-based cognitive therapy (MBCT) when compared to an active control.

**Method and analysis:**

This 1:1 randomised controlled trial will enroll 160 participants with PTSD symptoms in 2 arms (MBCT vs. Seeking Safety), with both interventions consisting of 8 weekly sessions lasting 2 h each week and led by certified instructors. Assessments will be conducted at baseline (T0), post-intervention (T1), and 3 months post-intervention (T2), with the primary outcome being PTSD symptoms measured by the PTSD checklist for DSM-5 (PCL-5) at T1. Secondary outcomes include depression, anxiety, attention, experimental avoidance, rumination, mindfulness, and coping skills. Both intention-to-treat and per-protocol analyses will be performed. Mediation analysis will investigate whether attention, experimental avoidance, and rumination mediate the effect of mindfulness on PTSD symptoms.

**Discussion:**

The proposed study will assess the effectiveness of MBCT in improving PTSD symptoms. The findings are anticipated to have implications for various areas of healthcare and contribute to the enhancement of existing intervention guidelines for PTSD.

**Trial registration number:**

ChiCTR2200061863.

**Supplementary Information:**

The online version contains supplementary material available at 10.1186/s12888-024-05840-x.

## Introduction

Post-traumatic stress disorder (PTSD) is a psychiatric condition triggered by experiencing or witnessing a traumatic event [1]. Some examples of traumatic events are sexual assault, domestic violence, childhood abuse, serious injury, illness, transportation accidents, natural disasters, or any stressful events that threaten personal lives or those of their close ones [2]. The major symptoms of PTSD include flashbacks of traumatic events, sleep disturbance, nightmares, rumination, negative thoughts, avoidance, and dysregulated hyperarousal [3]. While exposure to trauma is common, only a minority will develop and meet the full diagnostic criteria of PTSD [4]. Subthreshold PTSD refers to the condition in which only part of the symptoms are present clinically. While this condition does not fulfill the diagnosis of PTSD, it is found to be more prevalent in the community [4]. A study in 2014 conducted across 24 countries found that while 3% of participants met the diagnostic criteria of DSM-5 for PTSD, there was an additional 4.6% that met the criteria of subthreshold PTSD [4]. In general, these people experience symptoms that are less intense than full PTSD. However, their impairment is still significant to well-being and everyday life [5]. Zlotnick et al. found no significant differences in terms of functional impairment between patients with full PTSD and subthreshold PTSD, suggesting that people with subthreshold PTSD could experience negative impacts comparable to that of a full PTSD condition [6]. Other studies showed diminished social and family functioning [7], more days of absence from work [8], and a greater risk of suicidal ideation among subthreshold PTSD patients [9]. Besides, co-morbidity of mental health disorders, such as eating disorders, major depressive disorder, general anxiety disorder, and substance abuse are common among individuals with subthreshold PTSD [10]. Yet, some studies found that veterans with subthreshold PTSD did not utilize mental health services more than those with no PTSD, despite their significant impairment [11]. Another study on elderly with subthreshold PTSD found that they were more likely to perceive the care they received as inadequate [12]. These suggest that subthreshold PTSD might be easily overlooked by clinicians. Hence, subthreshold PTSD should be considered a condition that requires clinical attention and intervention.

Hong Kong is a multi-cultural society located in the southern part of China. In recent years, the COVID-19 pandemic and social unrest have adversely impacted the mental health of Hong Kong people [13]. Concerns have been raised mostly over major depressive disorder and PTSD [14, 15]. A survey based in Hong Kong conducted before the pandemic in 2014 revealed that 2.9% of participants developed current probable PTSD [16] The overall prevalence of experiencing trauma directly (i.e. responded happened to me on Life Event Checklist ) was 64.8%, while the rate increased further to 88.7% when indirect exposure is included (i.e. witnessed it or learned about it on Life Event Checklist) During the social unrest in 2019, the prevalence of suspected PTSD rose to 12.8% [17], and it increased further to 28.6% during the COVID-19 period in 2020 [18]. Such an increase in subthreshold PTSD was also found across different populations and cultures, which might be due to the COVID-19 pandemic around the globe [19–21]. With its increasing prevalence and the significant impairment it brings, subthreshold PTSD has become a global health problem that requires attention from both Hong Kong and the international community.

Most guidelines recommend manualized trauma-focused psychotherapies as the first-line treatment for PTSD [22]. On the other hand, medications could be used to assist treatment of PTSD symptomology [22]. Recommendations for PTSD medications might differ across guidelines [23]. In general, SSRIs outperform other medications and their efficacy has been evaluated in several studies [24]. However, meta-analysis studies found pharmacological intervention not as effective as psychotherapy [25]. A manualized psychotherapy contains guided standards for psychologists or psychiatrists to follow while ensuring that all trauma topics are well addressed. Over the years, several psychotherapeutic approaches have been developed to target PTSD. Examples are narrative exposure therapy, cognitive reprocessing therapies, eye-movement systematic desensitization and reprocessing and cognitive-behavioural therapy [22]. Psychotherapies mainly focus on the re-processing of traumatic memories, psychoeducation of coping skills and emotional regulation [26]. Seeking Safety is an evidence-based manualized psychological treatment. Its protocol was developed by Lisa Navajitis in the early 2000s that targets PTSD and substance abuse [27]. The core idea of Seeking Safety is to establish “Safety”. The word “Safety” here is an umbrella term that contains multiple elements: reducing suicidality, establishing safe relationships, taking good care of self, maintaining healthy habits etc [28]. . The whole protocol of Seeking Safety consists of 25 topics. These topics covers cognitive, behavorial and interspersonal domains. Examples of topics are “Safety”, “Compassion”, “Honesty”, “Asking for Help”, “Commitment”, “Creating meaning” etc. However, therapists can personalise and select suitable topics in the treatment manual. Over the past 20 years, various studies have been conducted showing that Seeking Safety could reduce PTSD symptoms significantly among different populations [29–31]. While the above treatments are evidence-based and recommended for PTSD, several concerns have been raised in various studies. First, there remains a certain level of dropout rate in trauma-focused psychotherapy, with the pool dropout rate at 16% [32]. Potential reasons for dropout might be treatment intolerance and symptom exacerbation due to traumatic reminders [33]. Besides, some psychologists have expressed concern about trauma-focused psychotherapy as this approach requires patients to unveil their trauma to the therapist [34]. Moreover, most of them retain the residual symptoms with the persistent diagnosis of PTSD after therapy [35]. Therefore, more alternative approaches are needed for managing PTSD.

In recent decades, there has been emerging evidence that supports mindfulness practice in improving individuals’ well-being and improving the outcomes of mental health problems [36]. Mindfulness-based interventions have been used extensively in psychiatric practice to treat various mental health problems, including major depressive disorder, general anxiety disorder, insomnia, addiction, substance abuse and chronic pain [37]. The idea of mindfulness originated from Buddhism and was later popularised in the West by Kabat-Zinn and his colleagues [38]. It involves bringing an individual’s focus to the present moment with an emphasis on a non-judgemental attitude [39]. It also cultivates acceptance and compassion for one’s thoughts, feelings, and bodily sensations [40]. In psychiatric practice, mindfulness could be incorporated into different psychotherapeutic approaches. The most studied mindfulness-based interventions are mindfulness-based cognitive therapy (MBCT) and mindfulness-based stress reduction (MBSR). MBCT combines mindfulness with cognitive behavioural therapy techniques and was initially developed to combat recurring depression, while MBSR focuses on coping strategies for pain and stress under chronic health conditions [41]. Both interventions encourage participants to practise mindfulness daily by offering home practice, mainly formal and informal practices [42]. Formal practices are offered through audio-recordings with instructions while informal practices require participants to integrate mindfulness elements into everyday life [43]. Currently, both MBCT and MBSR are considered evidence-based treatments and are efficacious for various mental health problems and psychiatric symptoms [44].

The evidence available suggests mindfulness-based interventions as a potential intervention for PTSD. In 2013, Kearney et al. showed that MBSR significantly reduced PTSD symptoms among 47 veterans in a pilot randomised control trial [45]. Similarly, King et al. conducted another randomised control trial with 37 participants by comparing MBCT with a treatment-as-usual group in combating PTSD symptoms [46]. Results showed that MBCT participants demonstrated significant improvement in both avoidance symptoms and PTSD cognitions. Another study by Kim et al. conducted on 28 nurses found that a 60-minute semi-weekly mindfulness stretching could significantly reduce PTSD symptoms, compared to an exercise control group [47]. The effect persists even until 16 weeks post-intervention. A randomised control trial among veterans with a larger sample size (*n* = 116) found that MBSR could significantly improve PTSD symptoms, compared to a group of present-centered focused therapy [48]. Multiple mechanisms have been suggested to explain how mindfulness could potentially improve PTSD symptoms. According to the model suggested by Lang, mindfulness emphasizes non-judgemental acceptance, allowing individuals to re-process traumatic pasts without extreme mood alternations [49]. In addition, mindfulness promotes the ability to focus on present moments, which helps patients shift attention away from arousal symptoms and other trauma-related stimuli. Lastly, mindfulness reduces avoidance and thought suppression by promoting openness to experiences. Other studies suggest that mindfulness breathing and meditation exercises promote relaxation which relieves uncomfortable feelings brought by PTSD [50]. Besides, neurobiological evidence showed that mindfulness might improve functional connectivity in multiple brain areas among PTSD patients [51]. These areas include the default mode network, frontoparietal network and salience network. A study conducted among veterans revealed increased activity in the medial prefrontal cortex to fearful cues after receiving a mindfulness-based exposure therapy, which might enhance top-down regulation of the limbic system and improve hyperarousal and intrusion symptoms [52]. This evidence supports mindfulness-based intervention as an effective treatment for PTSD and subthreshold PTSD.

Although preliminary evidence is available, there is no definite conclusion on whether mindfulness should be adopted as a formal intervention for PTSD [37]. First, most studies that support mindfulness-based intervention lack active control. The intervention group is often compared to waitlist control or no control which did not account for non-specific effects such as improvement over time [51]. Second, these studies are mostly conducted within a certain target group (e.g. veterans, women, police) with a relatively small sample size which limits the generalisability of findings. To our knowledge, no studies have previously targeted community adults. Third, most of these studies were conducted in the West. The effect of mindfulness-based intervention on reducing PTSD symptoms among Eastern cultures remains in doubt and still requires further exploration. Fourth, most of these studies lack a comprehensive secondary analysis that helps explain the mechanism of how mindfulness could reduce PTSD symptoms. Most of the models proposed are still theoretical and lack support. Hence, a well-designed study with a larger sample size and better control is needed.

Here, we propose a randomised control trial to evaluate the effectiveness of MBCT in reducing PTSD symptoms among Chinese adults in Hong Kong. The intervention will be compared to an active evidence-based control, Seeking Safety. Participants will be recruited from various community sources.

### Objectives and hypothesis

#### Objectives

- To evaluate the effectiveness of MBCT in reducing PTSD symptoms among Chinese adults in Hong Kong;
- To evaluate the effectiveness of MBCT in reducing depressive and anxiety symptoms, as well as promoting mindfulness and coping skills among Chinese adults with PTSD symptoms in Hong Kong;
- To evaluate if attentional control, experimental avoidance and rumination would mediate the effect of mindfulness on PTSD symptoms.

#### Hypotheses

- MBCT is more effective in reducing PTSD symptoms when compared to Seeking Safety, the active control group;
- MBCT is more effective in reducing depressive and anxiety symptoms when compared to Seeking Safety, the active control group.
- Attentional control, avoidance and ruminative thoughts are the mediators of MBCT in reducing PTSD symptoms in MBCT.

## Methods

### Study design

**Fig. 1:**
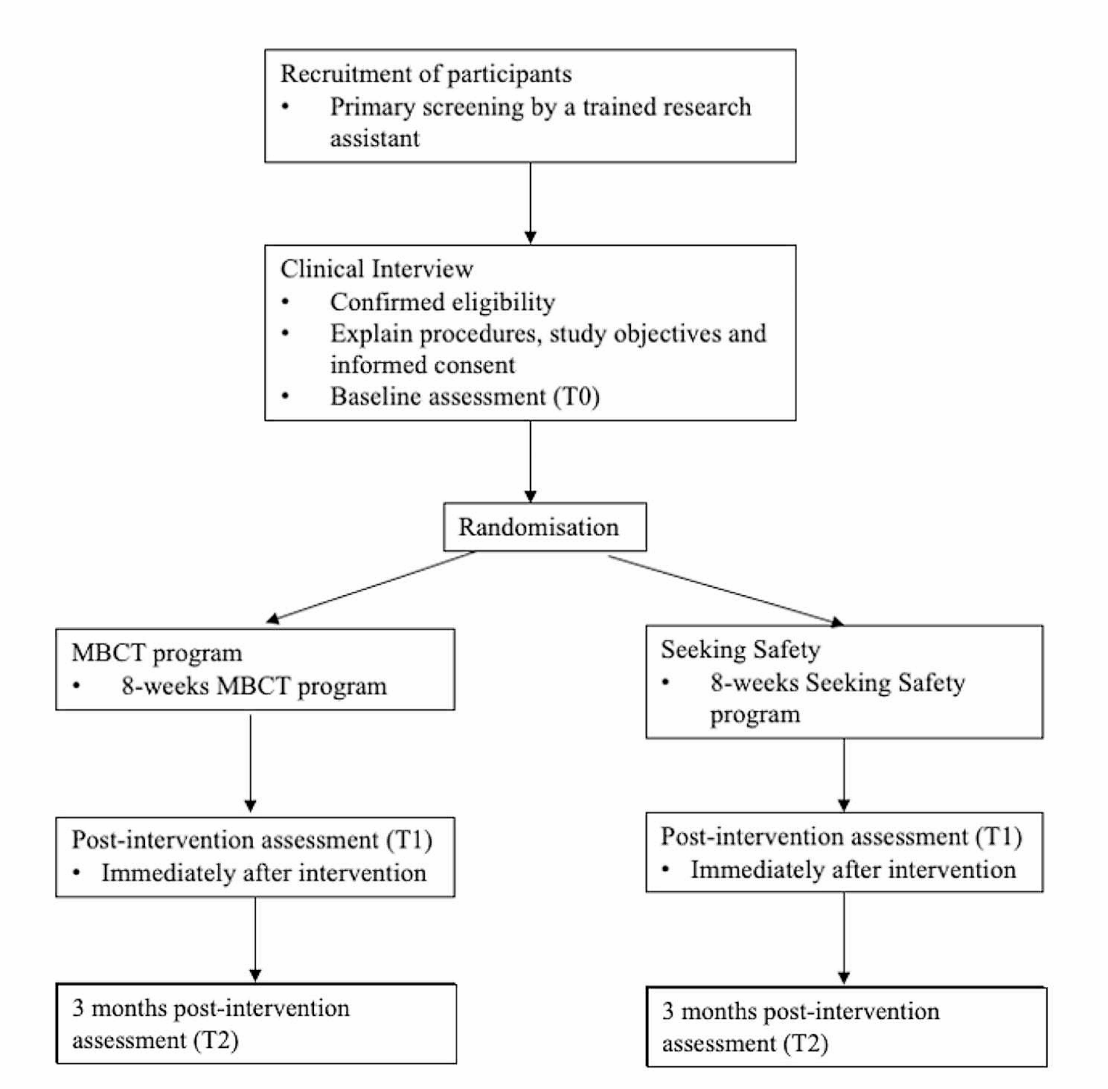
Flow of the proposed study. MBCT, mindfulness-based cognitive therapy

This project is a two-arm randomised controlled trial that evaluates the effectiveness of MBCT compared to an active control group, Seeking Safety. Assessments will be made at baseline (T0), post-intervention (T1) and 3 months post-intervention (T2) [48]. A summary of the study design is displayed in Fig. 1.

### Participants

Participants are Chinese adults aged 18 or above. They will be screened by the following inclusion and exclusion criteria. The inclusion criteria are (1) score 31 or above in the PTSD Checklist for DSM-5 (PCL-5), indicating the presence of significant PTSD symptoms [53], (2) PTSD symptoms must last for at least one month, and (3) If taking psychiatric medication, dosage must have been stable for 3 months. The exclusion criteria are (1) inability to understand Chinese, which is the language of all psychological sessions and materials, (2) possession of moderate to severe forms of diseases, either physical or psychological that renders participation in the study, and (3) previous participation in any structured mindfulness courses.

### Study setting and recruitment

This is a study with a multi-disciplinary collaboration including psychology, public health, psychiatry, and family medicine. Participants will be recruited from specialist psychiatric out-patient clinics, primary care clinics, psychiatry and clinical psychology departments of public hospitals of Hong Kong, community non-governmental organisations that provide psychiatric rehabilitation services and online support groups for patients with mental health disorders. Participants will either be referred by healthcare workers or apply via advertisements from these collaborators on a first-come-first-served basis. With reference to the inclusion and exclusion criteria, a primary screening will be done by a trained research assistant through telephone interviews. Those who pass the initial screening will meet a clinician to further confirm eligibility. Once eligible, a research assistant will further explain the procedures, study objectives and informed consent to the participants.

### Intervention: MBCT for PTSD

The intervention is MBCT with an established protocol utilised in prior studies for depression and anxiety. Minor adjustments are made to the protocol to tailor for the needs of trauma-sensitive individuals. The outline of the MBCT class is shown in Table 1. The MBCT is conducted in groups of up to 15 participants. It consists of 8 sessions with 2 h per week. Besides, to establish regular practices, a 45-minute homework per day is given to all participants. Audio recordings for home practice are provided and participants are suggested to document their practice through a given diary. Handouts and homework recordings are translated to Chinese by qualified health professionals, and are further face-validated by a group of expert mindfulness instructors. Instructors for MBCT are health professionals with formal training in mindfulness and certified to conduct MBCT. The training was referenced from the book, *Trauma-sensitive mindfulness: Practices for safe and transformative healing* [54]. This training will equip our instructors with the knowledge and skills to conduct mindfulness therapy for trauma-sensitive individuals. Several instructors instead of one will be recruited to ensure that the findings could be generalized.

**Table 1:** Outline of MBCT program modified for this study

Session	Theme	Session content
Session 1	Awareness and Automatic pilot – identifying and stepping out of automatic pilot	Group orientation Ground rules and introduction Self-care: grounding & alert techniques to keep one in the window of tolerance, aware of own conditions, respect own limitations… Mindful eating: Raisin exercise and review Body scan practice and review
Session 2	Living in Our Heads	Body scan practice Practice and homework review Thoughts, feelings exercises with respect to PTSD symptoms Pleasant events calendar Sitting meditation
Session 3	Gathering the Scattered Mind	5-minute seeing or hearing meditation Lying down yoga stretches and review Home practice review Standing stretch and breath, body meditation, and review 3 Steps breathing space and review Unpleasant events calendar
Session 4	Recognizing Aversion	Sitting meditation and review 5-minute seeing or hearing meditation Practice and homework review Territory of PTSD and depression Breathing space and review Mindful walking and review
Session 5	Allowing/Letting Be – acceptance of one’s experience	Sitting meditation (working with difficulties) and review Read Rumi’s Guest House Practice and homework review 3 Steps breathing space and review
Session 6	Thoughts Are Not Facts – seeing thoughts as mental events	Sitting meditation (working with difficulties) Practice and homework review Moods and thoughts exercise: mood and thoughts related/alternative viewpoints exercise 3 Steps breathing space and review Discuss breathing space as the ‘first step’ before taking a wider view of thoughts Discuss relapse signatures (Respond Wisely I)
Session 7	“How Can I Best Take Care of myself?”	Sitting meditation (includes working with difficulties) Practice and homework review Explore links between activity and mood Plan how best to schedule such activities 3 Steps breathing space as the ‘first step’ before choosing whether to take mindful action Discuss participants’ relationship with PTSD symptoms Discuss informal meditations during everyday life 3 Steps breathing space or mindful walking
Session 8	Maintaining and Extending New Learning	Body scan practice Practice and homework review Course review Discuss ways to continue mindfulness meditation beyond this programme End the class with a concluding meditation

### Active control: seeking safety

Seeking Safety is a manualised psychoeducational protocol developed by Lisa Najavitis [27]. The Chinese Version of the handouts has been translated by a group of experienced clinical psychologists from New Life Psychiatric Rehabitation Association in Hong Kong. Seeking Safety is a group intervention with a size of up to 15 participants. There are 8 sessions with a total of 2 h per week. Eight among twenty-five topics are selected for the sessions. They are “PTSD: Taking Back Your Power”, “Safety”, “Detaching from Emotional Pain (Grounding)”, “Asking for Help”, “Honesty”, “Setting Boundaries in Relationships”, “Compassion”, and “Taking Good Care of Yourself”. The selected topics were discussed with Najavitis to ensure the inclusion of the essential cognitive, behavioral, and interpersonal components of Seeking Safety. To encourage commitment and application of coping strategies discussed during the therapy sessions, participants could utilise the “Safe Coping sheet” and “Commitment to recovery form” if they found needed. Instructors are mental health professionals who have passed the certification test of Seeking Safety. Several instructors will be recruited to conduct the session to ensure generalisability.

### Intervention fidelity

Intervention fidelity of MBCT will be assessed by an independent health professional who is a mindfulness training expert. He/she will be provided with a simplified checklist developed from the Mindfulness-based cognitive therapy adherence scale (MBCT-AS). The MBCT-AS is a 17-item measure that assesses the treatment adherence of therapists to standard MBCT [55]. All the sessions will be audio-taped and 15% of them will be screened by the mindfulness expert to assess compliance with the MBCT protocol.

### Randomization, concealment and blindness

Participants are randomised to either MBCT or Seeking Safety on 1:1 ratio. Randomisation is stratified by gender and psychiatric medication use. Randomisation results will be generated by an independent statistician with a computer and sequences are sealed in opaque envelopes by another research assistant who is not involved in the study. Participants will be informed by the research team of their allocated group based on the results in the sealed envelopes prepared beforehand. Participants will not be allowed to switch to another intervention group. Statisticians who analyse the results will be kept blinded from the group assignment.

### Outcome measures

Demographic information will be collected at baseline, including their gender, age, education, income, marital status, medical history, medication, and types of traumatic events. Primary and secondary outcomes will be collected by a research assistant who is blinded to the group assignment at T0, T1 and T2 through questionnaires. In addition, participants from both groups will be asked to fill out a course evaluation at T1. Participants assigned to MBCT will fill out an extra section on their questionnaire indicating their mindfulness practice habits at T1 and T2. The primary and secondary outcome measures are shown below.

### Primary outcome

PCL-5 score at T1 is the primary outcome of this study. PCL-5 has four subscales and each corresponds to one DSM-5 symptoms cluster in PTSD, including intrusion, avoidance, negative alternations in cognition and mood, and alternations in arousal and reactivity [56]. It is a self-report measure that reflects the PTSD symptoms severity of participants on a Likert scale from 0 to 4, ranging from “not at all” to “extremely”. A total score could be obtained by simply summing up all 20 items. Participants who score equal to or higher than 31 are considered to display significant symptoms [53, 57]. The Chinese Version of PCL-5 is translated by Fung et al. with good internal consistency and reliability [58].

### Secondary outcomes

Patient Health Questionnaire-9 (PHQ-9) is used to measure the depressive symptoms of participants. It consists of 9 items total and a score could be obtained by simple summation. The cut-off scores for mild, moderate, moderately severe and severe depression are 5, 10, 15 and 20 respectively [59].

The Beck Anxiety Inventory (BAI) is used to measure the anxiety symptoms of participants [60]. It is a self-report measure with 21 items, consisting of the different symptoms that one might experience during anxiety. A total score could be obtained by adding up the items. The cut-off of 8, 16 and 26 represents mild, moderate and severe anxiety respectively [61]. The Chinese version of BAI has satisfactory internal consistency [62].

Attentional Control Scale (ACS) is used to measure attentional control [63]. It consists of 2 subscales which measure the 2 components of attention, including attention focusing and attention shifting. There are 20 items in total on a 4-point Likert scale, ranging from “almost never” to “always”.

Experimental avoidance is measured by Acceptance and Action Questionnaire-II (AAQ-II) [64]. The Chinese Version is validated with satisfactory reliability [65]. Psychological flexibility refers to the tendency to suppress or avoid negative thoughts, feelings, or emotions. It is a 7-point Likert scale ranging from 0 to 7, representing “never true” to “always true”.

Rumination is measured by the Rumination Response Scale (RRS). It consists of 22 items ranging from 0 to 4, representing “never” to “always”. The Chinese Version is validated with a higher internal consistency and reliability [66].

Mindfulness is measured by the Five-Facet Mindfulness Questionnaire (FFMQ). There are 20 items consisting of 5 subscales, which include observing, describing, acting with awareness, non-judgement and non-reaction. The Chinese Version of FFMQ has been validated by our team in prior studies, demonstrating a high internal consistency and reliability [67].

Coping Skills is measured by the 20-item Simplified Coping Style Questionnaire (SCSQ). The scale consists of 2 subscales, positive coping style and negative coping style. It is on a 4-point Likert scale, ranging from “never” to “very often”. It was originally developed from the Ways of Coping Questionnaire by Folkman and Lazarus. The SCSQ demonstrates good internal consistency and reliability [68].

### Statistical analysis

Statistical analysis will be conducted by a statistician blinded to the group assignment of participants. The study results will be presented according to the Consolidated Standards of Reporting Trial 10 (CONSORT-10) standards. The mean, median, standard deviation (SD), interquartile range (IQR) and 95% confidence interval (95% CI) will be presented in the data description. The analysis of covariance (ANCOVA) will be the primary analysis for the primary outcome (PCL-5) and other secondary outcomes (i.e., PHQ-9, BAI, ACS, AAQ-II, RRS, FFMQ, SCSQ), with T1 score as the outcome and T0 score as the covariate. Both intention-to-treat and per-protocol analyses will be performed. For per-protocol analysis, participants who attend more than 4 classes will be considered to have satisfactory compliance. Effect size estimates will be obtained by comparing the T0 and T1 scores mean and SD, both between and within groups. For the secondary analysis, we will apply linear mixed model (LLM) to compare changes over time for PCL-5 score and other secondary outcomes between the intervention group and the active control group. Missing data will be incorporated into the LLM based on full information maximum likelihood. Mediation analysis will be done using the Baron and Kenny mediation method and structural equation modelling (SEM). The analysis will explore if the effect of mindfulness on PTSD symptoms is mediated by rumination, experimental avoidance and attentional control.

### Sample size

G*Power (version 3.1.5) was used for calculating sample size. A previous study conducted on USA veterans with PTSD determined a between-group effect size of 0.59, comparing mindfulness-based intervention and present-centred therapy [48]. We hereby conservatively estimate the effect size of our study to be 0.5. Based on our previous studies on mindfulness-based intervention, we expect a 20% dropout rate at the end. Accounting for the dropout rate, and with a two-tailed $\alpha$error of 5%, 80% power, and 2 study groups, we will recruit 160 participants with 80 per arm.

### Ethical concern

Information about this study, including procedures, objectives, study period and randomisation arrangement will be provided to participants. Participants will be given time to consider whether or not they want to join the study. Once agreed, they will be asked to sign the informed consent with a duplicate copy kept by the research team members. Participants’ information and data will be kept in a password-protected computer and can be only assessed by responsible team members. Participants could choose to discontinue the study at any time. In case of adverse events, participants could seek help from the research team and the team will arrange for physicians certified in psychiatric practice or psychologists for further follow-up. All adverse events will be reported to the ethics committee immediately. The outcome of this study will be published in peer-reviewed journals and announced in public conferences according to the CONSORT-10 standards.

## Discussion

Mindfulness-based interventions are applied extensively in clinical settings for various mental health problems [37]. Subthreshold PTSD is a condition that displays significant PTSD symptoms that do not meet the full diagnostic criteria of PTSD. Nonetheless, these populations display clinically relevant symptoms that could persist for a very long time and adversely impact their life [5]. Preliminary evidence found mindfulness-based intervention effective in reducing PTSD symptoms in veterans. However, these studies are mainly conducted in the Western population, and the validity of these findings are limited by a lack of active control and small sample sizes.

The proposed study evaluates whether MBCT could reduce PTSD symptoms compared to the active control “Seeking Safety”. The two interventions are both group interventions with group size up to 15 participants, consisting of eight weekly 2-hour sessions in total. The findings of the proposed study have the potential to provide an alternative option for policymakers, clinicians and service providers to intervene clinically significant PTSD symptoms. Besides, it extends the current knowledge on how mindfulness works to improve the clinical outcomes for psychiatric problems.

The lack of mental health resources and services is common in many healthcare systems. While Hong Kong has one of the best healthcare systems, the waiting time for new cases to access secondary psychiatric care is very long. According to the Hospital Authority, from April 2022 to March 2023, the median waiting time for new but stable cases in outpatient psychiatric services ranges from 18 to 65 weeks across the seven clusters [69]. Besides, a survey of psychiatrists reported that patients in Hong Kong have low adherence to psychiatric medication mostly due to side effects and stigmatization [70]. Mindfulness-based intervention is an evidence-based treatment for various mental health problems and psychiatric symptoms. There is a lot of evidence that supports its efficacy and effectiveness. Such intervention could be conducted in groups with a relatively low cost, thus feasible in community settings. This could also be an alternative option for those who are unable to access secondary psychiatric care immediately. It is also an option for patients, including those with PTSD symptoms, who do not prefer medication in their first-line treatment.

There are several strengths in this study. First, the MBCT group is compared with an active control, which will control for non-specific effects. To our knowledge, this is the first randomised controlled trial with a large sample size compared to an active control conducted in the Eastern culture. If proven to be effective, the intervention we describe could be adopted as a formal intervention for people with PTSD symptoms in Hong Kong. Second, the study is conducted among community adults without specifically targeting a certain occupational group, which will have better generalization values. Third, mediators of the MBCT and outcomes will be investigated, which will deepen our understanding of the intervention mechanisms. Fourth, we will also do follow-up assessments until 3 months post-intervention, which allows us to evaluate both the short-term and sustained time effects of MBCT.

We anticipate several limitations in this study. First, participants randomised to either group might seek alternative interventions during the study, which might limit our comparison between the two groups. Although we explain and discourage participants from joining other interventions during the informed consent, it is not feasible and unethical to prevent participants from seeking other interventions, especially at the 3-months follow-up after intervention. For participants who seek other interventions, their interventions will be recorded and taken into consideration during the analysis. Second, participation in this study is completely voluntary, which might lead to a high drop-out rate. To compensate for that, we have accounted for the drop-out rate during sample size estimation, with reference to the drop-out rate of our previous randomised controlled trial studies regarding mindfulness. Third, our sample covers adults aged 18 and above with significant PTSD symptoms. Concerning a high co-morbidity of PTSD with other mental health disorders, the sample we recruit might be heterogeneous with different backgrounds which trade off the internal validity of the study. However, the study is designed in such a way to improve the generalisability of results, as subthreshold PTSD has a high co-morbidity with other mental health disorders [71]. To gain a better picture of the heterogeneity in our sample, we collect information regarding the participants’ mental health diagnoses and will consider integrating them for analysis.

Apart from the limitations mentioned, a major challenge would be recruiting participants. Since we target adults aged 18 and above, most of our participants will be the working class who might not have time to commit to all the intervention sessions and complete the weekly 45-minute homework in MBCT. This problem is common in studies conducted in busy cities such as Hong Kong. To further meet the needs of these participants, we arrange our classes at times convenient for the working population, including weekday nights or weekends daytime. We also provide interventions in different districts of Hong Kong to shorten the travelling time of these participants to encourage better commitment.

To conclude, the proposed study provides information on the effectiveness of MBCT in improving PTSD symptoms. It provides an alternative intervention option to patients, policymakers, and health service providers in combating PTSD, which is a healthcare problem in Hong Kong and also the international community. It is anticipated that the results will bring implications to different aspects of healthcare and improve current intervention guidelines for PTSD.

### Electronic supplementary material

Below is the link to the electronic supplementary material.

*Supplementary Material 1:* SPIRIT 2013 Checklist: Recommended items to address in a clinical trial protocol and related documents.

## Data Availability

The datasets generated and/or analysed during the study will be available from the corresponding author upon reasonable request following the completion of all primary publications derived from these datasets.
